# A smoke-free medical campus in Jerusalem: data for action

**DOI:** 10.1186/s13584-016-0080-9

**Published:** 2016-06-06

**Authors:** Itamar Feldman, Milka Donchin, Hagai Levine

**Affiliations:** Hebrew University-Hadassah Faculty of Medicine, Ein Kerem, 17 Habanai Street, Entrance A, Jerusalem, 9626430 Israel; Hebrew University-Hadassah Braun School of Public Health and Community Medicine, Ein Kerem, Jerusalem, Israel

**Keywords:** Smoke-free campus, Smoking policy, Tobacco control, Students, Medical education, Health professionals

## Abstract

**Background:**

Establishing smoke-free environments is a major component of tobacco control policy. The introduction of a smoke-free policy in medical campuses may serve as a role model for other educational and health institutions but little has been published about their prevalence or impact. In 2012, the Faculty of Medicine at Hebrew University–Hadassah in Jerusalem, Israel launched a smoke-free Medical Campus initiative. This study examined smoking behaviours, cigarette smoke exposure and attitudes towards the smoke-free campus policy among students and employees.

**Methods:**

Using a self-administered questionnaire, data was collected from medical, dental and pharmacy students, as well as employees of the school of pharmacy. We approached the entire target population in 2013 (*N* = 449), with a response rate of 72.5 % (*N* = 313).

**Results:**

The rate of smoking was 8.3 % (95 % CI 5.5–11.9 %). Most participants reported daily exposure or exposure several times a week to cigarette smoke (65.8 %). Overall, 98.0 % had reported seeing people smoke in open campus areas and 27.2 % indoors. Most participants supported the smoking ban inside buildings (94.2 %) but fewer supported (40.8 %) a complete ban of smoking throughout the campus, including outside areas. Only 18.4 % agreed that a policy prohibiting smoking was unfair to smokers. A multivariable analysis showed that support for a complete ban on smoking on campus was higher among non-smokers than for smokers (OR = 9.5, 95 % CI 2.2–31.5, *p* = 0.02).

**Conclusions:**

The smoke-free policy does not have total compliance, despite the strong support among both students and employees for a smoke-free medical campus. The data collected will assist policy makers move towards a total smoke-free medical campus and will aid tobacco control efforts in Israel and other countries.

## Background

Tobacco smoking is a major threat to public health and the most preventable cause of death [1]. Every year six million people die worldwide due to smoking, with over 600,000 dying of second-hand smoke (SHS) [2]. Tobacco use cost the American economy an estimated 289–332.5 billion dollars between 2009 and 2012 [1]. In Europe, the estimated cost of tobacco use in 2009 was 544 billion Euros [3].

It is estimated that 7,025 individuals died from smoking in 2014 in Israel - 793 of whom died of SHS - at an economic cost of 3600 million NIS [4]. Over the last few decades, smoking rates in Israel have dropped by 50 %, from 30–43 % in the mid 70’s to 18.7 % of the population aged 21+ in 2013 [5, 6] and 19.8 % in 2014 [7].

Establishing smoke-free environments is a major component of tobacco control policy. The International Public Health Convention of the World Health Organization (WHO) stipulates that the public should be protected from cigarette smoke exposure in public places as exposure to any level of smoking is harmful. Further, the only truly effective method of public protection is a complete ban of smoking in public areas. The convention notes that appropriate legislation, enforcement and ongoing evaluation are necessary components to the eradication of smoking in public places [8].

Implementation of smoke-free policies began in the USA in the 1970s with the introduction of designated non-smoking areas. By the 1990’s, legislation had expanded to include a total ban on smoking in specific public venues (such as airports). From the early 2000s, many countries started to introduce legislation that made smoking in public venues illegal. The scope and breadth of venue type is constantly being revised. The introduction of such legislation proved to be a turning point in the battle against smoking. Successful implementation of these policies proved that banning cigarette smoke from entire facilities is doable, is supported by the majority of the population and improves the social, economic and health status of the population [8, 9].

In Israel, smoke-free legislation commenced in 1983 with laws limiting smoking in certain public venues. Since then, smoking restrictions have been broadened to include a wide spectrum of public arenas – including both indoor and outdoor venues. These laws specify that smoke free public sites must post signs prohibiting smoking, cannot provide ashtrays in public areas, assign responsibility for breach of the ban on the owner of the establishment where the breach occurred, and have raised the size of fines [6].

As part of the efforts to reduce smoking in recent years, a no-smoking policy has become commonly accepted in American university campuses [10]. The means adopted included smoking restrictions, a smoke-free campus policy, quit smoking campaigns, and raising the price of cigarettes. Such interventions had a positive impact on students in reducing the number of smokers, changing attitudes towards students who smoke and smoking regulations. It was found that the smoke-free campus policy was more effective in reducing the number of smokers and prevalence of smoking in building foyers than media campaigns conveying information on the damage caused by smoking [11–13].

Health professionals have a central role in smoking prevention and cessation efforts, by providing counselling and smoking cessation advice and by setting a personal example. Successful implementation of a smoke-free medical campus policy may contribute significantly to a national tobacco control policy, serving as a role model for other public institutions. However, more needs to be known about how to successfully implement a smoke-free campus policy.

The Ein Kerem campus is part of the Hebrew University. The campus is physically separated from the other two big campuses of the university, and houses the Hadassah Ein Kerem Hospital, including Schools of Medicine, Dentistry, Pharmacy, Nursing and Public Health. Hadassah Ein Kerem has been a Smoke-free Hospital since 2000 [14] and a Health Promoting Hospital from 2009. In 2011, the Hebrew University adopted a Health Promoting and Sustainable Development policy. The Ein Kerem campus smoke-free policy was integrated into the framework of health promoting policies for Hadassah Medical Center and Hebrew University.

In May 2012, with the support of both students and staff, the Ein Kerem Smoke-Free Campus Declaration was signed by the hospital Director, university management and the Deans of the Faculties of Medicine and Dentistry. The declaration covers several commitments: prevention of exposure to SHS, complete prohibition of smoking within buildings, prohibition of onsite sale and marketing of cigarettes, supporting employee and student in smoking cessation, clinical research and training leadership in tobacco control efforts. Our target was to have a totally smoke-free campus within three years.

Despite all the steps described above, it seems that there is no full implementation and enforcement of smoke-free policies in Israel [6]. In Hadassah Ein Kerem, in 2004, four years after declaring Hadassah hospital a smoke-free hospital 41.4 % of participants still observed smokers in unauthorized places [14].

One year after declaring a Smoke Free Campus, there was a need to gather data on the current degree of smoke-free policy implementation and to evaluate the support for the current policy and future option of a total ban on smoking. The data will provide directions for an action plan to improve the implementation and support for smoke-free policy.

The purpose of the current study was to evaluate attitudes towards the smoke-free campus policy and their predictors among students and employees one year after policy implementation and the attitude toward total ban of smoking, smoking behaviour and cigarette smoke exposure as a basis for intervention to improve policy implementation as well as a reference for success in achieving our goal of a smoke-free campus by 2016.

## Methods

This was a cross-sectional study using a self-administered questionnaire at the Hebrew University Hadassah Ein Kerem campus. The target population for the study (*N* = 449) was the entire School of Pharmacy staff (*N* = 108) and all second year medical (*N* = 172), dental (*N* = 61) and pharmacy (*N* = 108) students.

We chose to sample the pharmacy staff for practical resons, as they are located in one building and are more accessible then the the other staff members (School of Medicine, School of Dentistry, School of Public Health).

The University’s board of Ethics and the Deans of the participating schools authorized the research.

Between May and August 2013, questionnaires were distributed amongst students attending lectures with expected high attendance rates. In November 2013, questionnaires were distributed individually to all School of Pharmacy employees.

The response rate for students was 60 % (Medicine), 66 % (Dentistry), and 80 % (Pharmacy). The response rate among employees was 92 %. The overall response rate was 72.5 % (*N* = 313).

The questionnaire comprised: [1] demographic details: age, gender, sector (student/employee), study track (Medicine/Dentistry/Pharmacy) and marital status (married/unmarried) [2] smoking status (current smoker/ex-smoker/never smoked) [3] attitudes towards a smoke-free campus policy, based on the degree of agreement to 5 statements based on a Likert scale of 1 (do not agree) to 5 (strongly agree) [4] degree of exposure to cigarette smoke: frequency with which they witnessed smokers both indoors and in outdoor areas of the campus, frequency with which they could smell cigarette smoke on campus, and frequency of being in the vicinity of smokers in outdoor areas of the campus; and [5] awareness of the existence of the smoke-free campus policy (aware/unaware/uncertain). For univariate analysis, *χ*^*2*^ test or Fisher’s exact tests was used and for multivariate analysis, a logistic regression was performed. All statistical analysis used the SPSS Statistic program by SPSS Inc., version 17.

## Results

The study included 313 individuals of whom 214 were students, either from the Faculties of Medicine (102), Dentistry (43) or Pharmacy (69), and 99 employees of the School of Pharmacy. Of all participants, 35.3 were males and 64.7 % females; 10.8 of students and 59.8 % of employees were married. The average age of students was 23.6 (SD = 2.7) and the average age of employees was 40.0 (SD = 14.5) [Table 1].

**Table 1:** Demographic characteristics of the study population

	Students *N* = 214	Employees *N* = 99	Total *N* = 313
Age - mean (SD)	23.6 (2.7)	39.8 (14.5)	28.7 (11.3)
Gender (%)			
Male	36.0	33.7	35.3
Female	64.0	66.3	64.7
Family status (%)			
Married	10.8	59.7	25.7
Unmarried	89.2	40.2	74.2
Study Track (%)			
Medicine	47.6		
Dentistry	20.1		
Pharmacy	32.2		

### Smoking habits

Only 8.3 % (95 % CI 5.5–11.9 %) of participants – students and employees alike – were smokers. Employees were more likely to be ex-smokers (22.2 %) than students (13.6 %) were. Current smoking and past smoking were more prevalent among men (RR = 1.35 and RR = 1.9, respectively, *p* = 0.02) [Table 2]. Married participants were twice more likely to be ex-smokers than unmarried participants (RR = 1.9, *p* = 0.016). Within each age group, smoking rates were higher for unmarried than married participants. The highest rate of smoking was among unmarried individuals in the 24 – 40 year age group (15.3 %).

**Table 2:** Smoking habits of the study population

	N	Past smokers (%)	Smokers (%)	*P*-value*
Total	313	16.3	8.3	
Sector				N.S^a^
Student	214	13.6	8.6	
Employee	99	22.2	8.2	
Gender				0.02
Male	110	23.6	10.0	
Female	202	12.4	7.4	
Marital status				0.02
Unmarried	231	13.0	9.5	
Married	80	26.3	5.0	
Age group				0.03
< 24	144	9.7	6.3	
40–24	127	22.0	12.6	
>40	38	23.7	2.6	
Study Track				N.S^a^
Medicine	102	12.7	6.9	
Dentistry	43	16.3	11.6	
Pharmacy	69	12.0	8.7	

*p-value for differences between the groups (regarding smoking status)

^a^N.S - Not significant

### Exposure to cigarette smoke

27.2 % of study participants reported seeing people smoke inside campus buildings on a weekly or monthly basis. Such seeing were associated with age, sector, marital status, smoking status and study track. Students were more likely to report seeing people smoke within campus buildings than employees (37.5 % vs. 15.4 %). Similar findings were found for unmarried participants (31.8 % vs. 14.7 %). For both sectors (students and employees), non-smokers were more likely to report having seen people smoke inside campus buildings than ex-smokers or current smokers (30.0 % vs. 25.0 % & 7.7 % respectively) [Table 3].

**Table 3:** Smoke exposure by campus area (inside vs outside)

	Observed smokers in enclosed areas (%)				Observed smokers in open area (%)			
	N	Weekly	Monthly	Never	N	Weekly	Monthly	Never
Total	291	14.8	12.4	72.9	313	82.5	15.5	2.0
Status								
Student	200	17.5	15.0	67.5	200	79.5	18.0	2.5^a^
Employee	91	8.8	6.6	84.6	97	88.7	10.3	1.0
Smoking status								
Current	26	7.7	0.0	92.3	26	84.6	11.5	3.8
Past	48	20.8	4.2	75.0	48	93.8	2.1	4.2
Never	217	14.3	15.7	70.0	223	79.8	18.8	1.3
Age group								
< 24	133	21.1	15.0	63.9	134	82.8	14.2	3.9^a^
24–40	119	10.9	9.2	79.8	124	83.1	16.1	0.8
> 40	36	5.6	11.1	83.3	36	80.6	16.7	2.8
Gender								
Male	104	12.5	13.5	74.0^a^	106	84.0	12.3	3.8^a^
Female	187	16.0	11.8	72.2	190	82.1	16.8	1.1
Marital status								
Unmarried	214	16.4	15.4	68.2	217	82.0	15.2	2.8^a^
Married	75	10.7	4.0	85.3	78	83.3	16.7	0.0

^a^No significant difference was found between groups

In all, 82.5 % of the study population reported seeing people smoke in outdoor campus areas at least twice a week. Among all those who reported observing smokers in outdoor campus areas (*N* = 255) only 13 % indicated that it was in a designated smoking area. Smoking outdoors was most commonly observed in the campus inner courtyard (25.5), entrance plaza (24), bench area (14.5) and building entrances (5.5 %), areas where smoking is forbidden.

65.7 % of the study participants reported exposure to a smoky environment at least once a week. Current smokers had the highest probability of being in a smoky environment compared to ex- and non- smokers (96.2 % vs. 74.0 % & 60.2 % respectively, p-value <0.01). Unmarried and participants aged 24–40 had a higher probability of being in a smoky environment than their counterparts, irrespective of smoking status (OR =2.13 and 3.98 respectively).

### Awareness of and attitudes towards the smoke-free campus policy

In all, 46.6 % of study participants had heard of the smoke-free campus policy, 40.1 % had not heard of it, and 13.4 % were not sure.

Most participants agreed that smokers should smoke only in outside designated areas (93.9 %), and that all campus buildings should be completely smoke-free (94.2 %). The strongest predictor of agreement to all statements expressing total prohibition of smoking in campus buildings is being a non-smoker. Those aware of the smoke-free campus policy were more likely to agree with the statement that smokers should smoke only in outside designated areas. Those reporting exposure to cigarette smoke were significantly more likely to agree that all campus buildings should be smoke-free [Table 4].

**Table 4:** Factors associated with agreement with statements regarding smoke-free policy, multivariate logistic regression

			Percentage of agreement (%)	OR (95 % CI)	*P*-value
Smoking on campus should only be permitted in designated smoking areas	Smoking status	Current	73.1	1.0	^a^ < 0.01
		Past	82.4	2.2 (0.6–7.3)	
		Never	98.7	39.4 (8.8–176.7)	
	Awareness of the smoke-free campus policy	Unaware/uncertain	90.9	1.0	<0.01
		Aware	97.2	5.3 (1.6–17.9)	
All campus buildings should be smoke-free	Smoking status	Current	84.6	1.0	
		Past	84.3	0.8 (0.2–3.0)	^a^<0.01
		Never	97.4	7.0 (1.8–27.5)	
	Smelling cigarette smoke	Did not smell	90.8	1.0	<0.01
		Smelled	97.0	4.2 (1.4–12.8)	
The smoking policy ban is unfair to smokers	Smoking status	Never	14.5	1.0	^b^0.02
		Current	30.8	2.4 (0.7–7.6)	
		Past	29.4	3.4 (1.4–8.5)	
	Track	Pharmacy	30.9	1.0	<0.01
		Medicine	13.0	0.3 (0.2–0.7)	
		Dentistry	11.6	0.3 (0.1–0.8)	
All campus areas should be smoke-free	Smoking status	Current	7.7	1.0	0.02
		Past	25.5	3.1 (0.6–15.5)	
		Never	47.8	9.5 (2.2–31.5)	
	Observing smokers in open area	Didn’t observe	34.3	1.0	<0.01
		Observed	55.1	2.1 (1.2–3.6)	
Smoking should be restricted to the campus gates, up to a distance of 10 m	Smoking status	Current	11.5	1.0	^a^ < 0.01
		Past	31.4	2.9 (0.8–11.3)	
		Never	47.8	6.8 (2.0–23.5)	
	Family status	Unmarried	38.1	1.0	<0.01
		Married	53.0	2.0 (1.2–3.4)	

^a^Significant difference was found only between current and never smokers

^b^Significant difference was found only between past and never smokers

Only 30.8 of smokers, 29.4 of ex-smokers and 14.5 % of non-smokers agreed that banning smoking is unfair to smokers. Higher rates of students in the school of Pharmacy agreed with the above statement than students of Dentistry and Medicine (30.9 compared to 11.6 and 13.0 %). The most significant predictor of agreement with this statement is being an ex-smoker, and being a pharmacy student [Table 4].

Whilst the majority agreed that smoking should be limited to outside designated areas, less than half also supported a total prohibition of smoking on campus (40.8 %) with another 42.2 % agreeing that smoking should be restricted to the campus entrance (a distance of 10 m from building entrances). The strongest predictor of agreement that smoking should be absolutely prohibited throughout the entire campus was being a non-smoker (OR 6.8 and 9.5 for two statements that express this agreement, Table 4). Observing smokers in open area significantly predicts agreement that the entire university campus should be smoke-free (OR 2.1), while the status of being married significantly predicts agreement that smoking should be restricted to campus entrances (OR 2.0) [Table 4].

## Discussion

The purpose of this study was to evaluate the implementation of a smoke-free policy in a medical campus, by assessing attitudes towards the smoke-free campus policy, smoking habits and cigarette smoke exposure. Our findings further directed policy formation and implementation of the smoke-free Ein Kerem Campus. Less than 10 % of students and employees are current smokers but nearly two thirds reported being near smokers on a daily or almost daily basis. Strong support amongst both students and employees was found for a smoke-free medical campus policy. However, one year post-implementation, less than half the target population were aware of its existence and smoking in unauthorized areas was still prevalent.

There is little data available regarding smoke-free Medical campuses. However, several studies have reported findings for smoke-free universities. An online survey conducted in a large Australian university, one year after becoming a smoke-free campus, found lower smoking rates than in the general population, negative attitudes toward cigarette smoking and an overwhelming support for a smoking ban inside university buildings, but less support for a total smoking ban on the campus [13]. Another web-based survey among students in Pacific Northwest public university (USA) found a high degree of support (76 %) for a smoke-free campus policy and that current smoking as well as past smoking was strongly associated with opposition to a smoke-free policy [15]. Overall, a smoke-free campus policies have been found to be effective in reducing smoking rates in general and smoking in building foyers areas in particular [11–13].

The data gathered here provides a benchmark for the impact of the smoke-free policy on smoking behaviour and attitudes and will provide a comparison for future study. It further indicates where the focus of further intervention should be: increasing awareness and support to the policy, engaging ex-smokers and enforcing ban in areas such as the inner courtyard.

The smoking rate among health professional students at Ein Kerem campus in 2013 was much lower than that found in the general population (8.6 %, vs 19.8 %). Rates were lower for both genders, but particularly so for males (10 % vs 27 %) than for females (7 % vs 13 %) [5]. The smoking rates found here are much lower than the global smoking rate for health professional students in 2005–2008 (22.4 %) [16], and comparable to the USA in 2010 (6 %) [17]. In a single hospital study carried out in 2013, smoking rates among physicians was 16.7 % [17] compared to only 6.9 % found here for medical students. However, a larger scale study amongst Israeli physicians and other medical university campuses in Israel would be required in order to confirm that today’s medical students smoke less than physicians.

A study conducted in 2007 among dentistry students at Ein Kerem campus found a smoking rate of 17.0 % [18]. The lower smoking rates in our study (11.6 %) are consistent with the decline in smoking rates found among the general population of Israel between 2007 and 2013 (23.2 and 18.7 % respectively). The steps taken at Hadassah Medical Center and the campus since 2000, declaring the hospital a smoke-free area [14], and later signing the Ein Kerem Smoke-free Campus Declaration in 2012 may also have contributed to this drop in smoking prevalence. The current study highlights the need for Tobacco surveillance data for health professional students in Israel, as described in the Global Health Professions Student Survey (GHPSS) [16].

It was shown previously that non-smoking physicians have a greater influence on people trying to quit smoking than smoking physicians [19]. Therefore, a decrease in the rate of smoking among physicians would improve smoking cessation rates in the general population [20].

The higher smoking rate among males correlates with earlier studies demonstrating that male medical students generally have higher smoking rate [21].

The difference in smoking rates between men and women (10.0 % compared to 7.5 %) was comparable to that of health professional students in Europe and the USA [16, 21] but lower than the difference observed in the Israeli general population (27.3 % vs 12.6 %) [4].

Despite the smoke-free policy and consensus among students and employees that campus buildings should be smoke-free, and that smoking should only occur in designated areas, significant levels of exposure to tobacco smoke on campus were reported. 27 % of participants reported seeing people smoke within the buildings at least once a month and that the majority of participants observed smokers in open spaces, and in most cases, not in designated smoking areas. These findings suggest that enforcement of smoke-free campus policy should be stronger.

The percentage of students and employees (65.2 %) who spend time with smokers is comparable to the rate of second hand smoke in Israel 71.3 % [14]. Students and employees 24 to 40 year olds, ex-smokers and unmarried individuals are more likely to be exposed to cigarette smoke.

Consistent with earlier studies [21], ex-smokers were more likely to show more support of various components of smoke-free policy than smokers, but show less support than non-smokers. Although ex-smokers are not directly affected by such policy, it appears that they still identify with smokers and are more reluctant than non-smokers to promote a total smoking ban. Current smokers were less supportive of the smoke-free policy, as was found in other studies [22]. Only 18 % of the study population believed that smoke-free policy is unfair toward smokers.

A total ban on smoking (including in designated areas) is more effective than a partial ban in lowering smoking percentage and SHS exposure [23–27]. Therefore, as public health planners, we should consider expanding the smoke-free policy to a total ban on smoking.

The smoke-free hospital policy became common in the USA in the early 1990s and in Europe in the early 2000s [28]. Initially it was a voluntary recommendation, part of a general strategy to promote a general smoke-free policy and only banning smoking in indoor areas for staff members. Later, with the accumulation of evidences showing that a more restrictive smoke-free policy is more efficient [29], a new movement to promote completely smoke-free hospital campuses, which extended smoking bans to outdoor areas, emerged [30, 31] and, in 2008 in the USA, over 45 % of hospitals reported they had extended smoke-free policies to include outdoor spaces [30].

As opposed to a partial ban on smoking, whereby smoking is allowed in designated areas, a total ban on smoking requires intervention that could be considered by some as unjustified interference in their freedom of choice, even when it comes to leading unhealthy lives.

The assessment model for justification of intrusive lifestyle intervention sets a principle of support, which requires policy planners to ask two questions: Is the time ripe for coercion and is there enough support for the intervention? Positive answers to these questions are necessary to justify the intervention and to assess whether we will achieve proper implementation and enforcement or not [32].

Over 90 % of the participants agreed that indoor areas should be smoke-free and that smoking should be restricted wholly and solely to designated smoking areas. However, less than 50 % agreed that all campus areas should be smoke-free and smoking prohibited in campus entrances. This preference for restrictions rather than a total ban has been reported in other studies. A survey conducted in England (2011) among hospital employees and medical students found that only 40 % favoured a complete ban compared to 57 % who favoured restrictions only [33]. Another survey conducted in Australia (2013) found similar results: 91.3 % agreed that the campus should be smoke-free inside all buildings whilst only 60.8 % agreed that the whole campus (indoors and outdoors) should be smoke-free [13].

Compared to a survey conducted at Ein Kerem campus among students of dentistry in 2006, a total ban policy has gained in support. In 2006, 51 % agreed to the statement supporting a total prohibition on campus [18], compared to 72 % in the current study. Introduction of a pro-active policy to reduce smoking in recent years on the campus and in the adjacent Hadassah Ein Kerem Hospital may explain the increase in support.

Support for all statements was stronger among non-smokers than ex-smokers and current smokers. However, even among smokers, the majority agreed that indoor areas should be smoke-free (94.2 %), and that smoking should be restricted to outdoor designated smoking areas (93.9 %). Similar findings were found in a national American study [18]. On the other hand, when it comes to a total smoking ban on campus, smokers show less support. This pattern of limited support among smokers is consistent with earlier studies [34, 35]. Investment in advocacy for for a total smoking ban is needed in order to implement the new policy.

Awareness of the smoke-free campus policy was strongly associated with support of a smoke-free policy in our study, as was found in other studies [12]. Less than 50 % were aware of the smoke-free campus policy.

These findings emphasize the need of a comprehensive intervention aimed to increase awareness of the smoke-free campus policy among students.

The choice of target population and their response rate could factor as limitations of the study. We targeted second year students and employees of the School of Pharmacy. Results for other years or other schools in the campus may differ. Whilst the response rate was high (72.5 %), our results could still be biased if those who did not participate differed in their smoking behavior or attitudes from participants. We minimized the possibility of selection bias by an objective presentation of the study and support of deans. Refusal was minimal and the main reason for non-participation was not being present in class or office.

## Conclusions

We found that whilst there was strong support for the smoke-free Ein Kerem campus policy, its adherence was only partial. Disparities still exist between support for restricting smoking and its actual realisation. Further, a complete ban is not yet fully supported by the majority of the student body or staff. The data gathered and tools developed in this study will serve both as a basis for planning and determining the impact of further interventions for this campus and others in Israel and abroad.

## Abbreviations

GHPSS, Global Health Professionals Student Survey; SHS, Second Hand Smoke.
